# Swimming and feeding of mixotrophic biflagellates

**DOI:** 10.1038/srep39892

**Published:** 2017-01-05

**Authors:** Julia Dölger, Lasse Tor Nielsen, Thomas Kiørboe, Anders Andersen

**Affiliations:** 1 Technical University of Denmark, Department of Physics and Centre for Ocean Life, DK-2800 Kgs. Lyngby, Denmark; 2 Technical University of Denmark, National Institute of Aquatic Resources and Centre for Ocean Life, DK-2920 Charlottenlund, Denmark

## Abstract

Many unicellular flagellates are mixotrophic and access resources through both photosynthesis and prey capture. Their fitness depends on those processes as well as on swimming and predator avoidance. How does the flagellar arrangement and beat pattern of the flagellate affect swimming speed, predation risk due to flow-sensing predators, and prey capture? Here, we describe measured flows around two species of mixotrophic, biflagellated haptophytes with qualitatively different flagellar arrangements and beat patterns. We model the near cell flows using two symmetrically arranged point forces with variable position next to a no-slip sphere. Utilizing the observations and the model we find that puller force arrangements favour feeding, whereas equatorial force arrangements favour fast and quiet swimming. We determine the capture rates of both passive and motile prey, and we show that the flow facilitates transport of captured prey along the haptonema structure. We argue that prey capture alone cannot fulfil the energy needs of the observed species, and that the mixotrophic life strategy is essential for survival.

Small plankton form an essential part of the marine ecosystem. Such organisms face the challenge of living in a light- and nutrient-limited environment, while being exposed to flow-sensing predators. Many unicellular flagellates in the size range from 2 to 50 micrometer are mixotrophic and use a combination of photosynthesis, dissolved nutrient uptake, and prey capture to access resources^1^,^2^. Despite the increase in predation risk due to the induced flow disturbances, they must swim to reach light and food and create feeding currents that enhance prey capture and nutrient uptake. They do so by means of cilia and flagella in different numbers and with different positions, lengths, and dynamics^3^,^4^,^5^. This diversity in flagellar arrangements suggests different strategies with trade-offs, since not all functions can be optimized simultaneously. Biflagellates with two left-right symmetrically arranged flagella, such as the well-studied algae *Chlamydomonas reinhardtii*, are an abundant and successful flagellate form^4^,^6^. By tuning the flagellar arrangement and beat pattern, biflagellates can arrange the thrust forces in front of the cell (puller), equatorially (neutral), or behind the cell (pusher)^7^. What are the advantages and disadvantages of different flagellar arrangements and beat patterns in mixotrophic biflagellates, and to what extent are these archetypical, multi-functional organisms optimized for swimming, predator avoidance, and prey capture? To answer these questions we focus on two mixotrophic, biflagellated species of haptophytes with different morphologies, kinematics, and feeding strategies (Methods, Fig. 1, and Supplementary Videos S1 and S2). *Prymnesium polylepis* has long flagella that move in an undulatory fashion and it feeds on small prey captured on the long and slender haptonema that emerges from the cell front. The feeding process involves capture, transport along the haptonema, and delivery of prey to the ingestion site at the opposite end of the cell^8^ (Fig. 1c–f). *Prymnesium parvum* feeds on much larger food items, and even performs micropredation on fish using toxins^9^,^10^. Organisms of this species do not have an apparent use of the haptonema and exhibit a short haptonema and short flagella moving with a ciliary beat.

The flow around an organism produced by its flagellar motion is important for all essential functions^3^. It reveals information about swimming, power consumption, feeding currents, and exposure to flow-sensing predators^11^,^12^,^13^. Idealized viscous flow models can be used to examine the hydrodynamics around a microswimmer^3^,^14^. Models representing a swimmer just by a few point forces on the fluid are able to describe the flow far from the organism^11^,^15^,^16^, whereas the flow close to the organism is poorly represented since such models fail to describe the boundary conditions at the cell surface. Examples of models suited for the description of near cell flows are the squirmer model used for ciliates^17^,^18^ and the Oseen model used for copepods and uniflagellates^12^,^19^,^20^,^21^. The effect of different flagellar arrangements and beat patterns in biflagellates has previously been investigated with focus on swimming and nutrient uptake^22^, swimming and flagellar synchronization^23^,^24^, and quiet swimming^13^. Hydrodynamic interactions between cell and flagellum play an important role for propulsion, and therefore provide one reason for models to take into account the no-slip boundary condition at the cell surface^25^.

Here, we investigate how different flagellar arrangements and beat patterns in biflagellates affect swimming speed, flow disturbance, and prey capture. We examine how far each of these essential functions is optimized in mixotrophs. The flow fields of the two characteristically different haptophyte species are visualized using micro particle image velocimetry (Methods). To explore the influence of the flagellar arrangement, we build on the Oseen model and develop an analytical biflagellate model consisting of two point forces in the vicinity of a spherical body with no-slip boundary. The model captures the essential features of the observed flow. With the model we quantify the time-varying and the time-averaged near cell flows around the two species and we find optima for swimming, predator avoidance, and prey capture.

## Results

### Flagellar arrangements, beat patterns, and flow fields

The two species show characteristic differences in their flagellar arrangements and beat patterns and in the resulting flow fields. *Prymnesium polylepis* has long flagella that beat in an undulatory mode with travelling waves that move down the flagella (Fig. 2a–d, Table 1, Supplementary Video S3). The phase shift between the two flagella does not show a clear pattern and varies across individuals and over time. The swimming speed is constant. Behind the organism, large, mainly transversal time-varying flows are formed around the beating flagella. The time-dependent flow is qualitatively different for *P. parvum*, that has short flagella and swims with an unsteady ciliary beat pattern leading to large variation in swimming velocity during the beat cycle (Fig. 2e–h, Table 1, Supplementary Video S4). In each beat phase one can note symmetrically arranged patches with high flow speeds, the flow directions and positions of which follow roughly the dynamics of the flagella end segments. *Prymnesium parvum*, as observed, mainly swims with a synchronous beat, which is interrupted by periods of asynchronous “tumbling” motion. The beat pattern, flow fields, and swimming velocity variation during the beat cycle of *P. parvum* resemble roughly those of *C. reinhardtii*^15^,^22^,^26^. However, the beat pattern differs in the characteristic backwards bending of the flagella during the power stroke (Fig. 2e,f).

To explore the effect of the flagellar arrangements and beat patterns we developed a model for freely swimming biflagellates. The cell body, modelled as a no-slip sphere, is propelled by two left-right symmetrically arranged point forces acting on the water (Fig. 3). The model is based on Oseen’s solution of the Stokes equation for the flow due to a single point force next to a stationary sphere^27^,^28^. We neglect the friction on the two flagella compared to the friction on the cell. One can think of the cell and the two point forces as being connected by a thin, rigid, and frictionless scaffold^23^,^24^. To obtain the flow around the freely swimming model biflagellate we use the linearity of the Stokes equation when superposing the flow due to each of the point forces and the flow due to the sphere towed with the translational velocity calculated from the force balance^19^. The forces on the model flagellate are the thrust force −(**F**_1_ + **F**_2_), the force **K**_1_ + **K**_2_ due to the flow produced by the two point forces on the water, and the Stokes drag **D** = −6*πμa***U**, where *μ* is the dynamic viscosity and **U** the velocity of the translational motion of the cell^19^. We therefore have the force balance on the flagellate


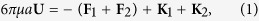


and since the forces **K**_1_ and **K**_2_ are known analytically^28^, we can determine the translational velocity of the cell.

The time-averaged flows close to and in front of the cell body are well represented for both haptophyte species by the biflagellate model with backwards directed point forces at characteristic positions, which we roughly estimated by visual comparison of the model flow fields with the measured flow fields around one organism of each species (Fig. 4, Supplementary Fig. S5, and Table 1). The time-resolved flow fields around *P. polylepis* show an extended, time-varying and often asymmetric force distribution along the flagella. This makes the simple biflagellate model for this organism only applicable for the time-averaged flow. To model the time-dependent flow around *P. parvum*, we used the rapidly moving, tracked flagellar ends to model the thrust force positions, since we assume that they contribute significantly to the force production. Also, the flagellar end segments appear to be transversely oriented to the trajectories in the main part of *P. parvum*’s beat cycle, and we therefore applied forces of constant magnitude acting tangentially to the trajectories. We estimated elliptic trajectories with the downwards power stroke further away from the body than the upwards return stroke. The model biflagellate is able to swim due to the difference in the drag on the cell body between the power stroke and the return stroke (Fig. 5). Furthermore the forces in the transverse direction towards and away from the cell contribute more than 20% to the forward drag due to their favourable arrangement below and above the equator. The flow fields of the simple model strongly resemble the measured flow fields of *P. parvum* (Figs 2e–h and 5a–d). We conclude that the variation of the thrust force position during the beat cycle can play a large role for the swimming of biflagellates. Similar findings on the role of thrust force positions were made on *C. reinhardtii* using a numerical singularity model^25^ and a three-sphere model^24^.

### Swimming speed and flow disturbance

Swimming speed as well as flow disturbance depend characteristically on the flagellar arrangement. The swimming speed *U* can be determined theoretically from the force balance on the flagellate (1). The force **K**_1_ + **K**_2_ is proportional to the magnitude of the point forces *F* = |**F**_1_| = |**F**_2_|, and it depends strongly on the radial force position *R* and the angular force position *θ* (Fig. 3) [28, p. 88, eq. (3.3.26)]. In the biflagellate model with thrust forces in the positive *z*-direction the force expression simplifies to


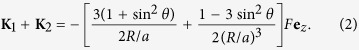


We therefore analytically obtain the swimming speed as


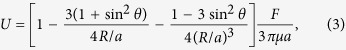


where the highest swimming speed *U*_∞_ = *F*/(3*πμa*) for constant thrust force magnitude is obtained when the thrust forces are placed far from the cell body and the model reduces to the flow due to a towed sphere. By arranging the thrust forces equatorially the swimming speed reaches a maximum for constant thrust force magnitude and distance. The speed for this arrangement is significantly higher than for the corresponding puller or pusher when the thrust forces are close to the cell body (Fig. 6a). An optimal equatorial flagellar arrangement is found for *P. parvum* in time-average, whereas *P. polylepis* with its estimated angular force position appears to have a flagellar arrangement that is not optimized for fast propulsion.

To reduce the risk of detection by flow-sensing predators the haptophytes should make their disturbance zones as small as possible^11^. How does the size of the disturbance zone vary with force positions for a fixed swimming speed? It has been shown that for detection of prey by a larger predator absolute flow velocities matter as opposed to spatial and temporal derivatives, which in prey can trigger escape from predators^29^. The mean threshold speed was found to be 40–50 *μ*m s^−1^ for prey detection by the ciliate *Mesodinium pulex*, which feeds on similar-sized prey as haptophytes^30^, and the threshold value 40 *μ*m s^−1^ has been measured for the copepod *Oithona similis*^29^. Thus we shall use 40 *μ*m s^−1^ as a reasonable threshold speed estimate for the detection of haptophytes. We define the disturbance zone as the volume inside which the flow speed exceeds 40 *μ*m s^−1^. The maximum disturbance area in the *xz*-plane during the beat cycle for *P. polylepis* and *P. parvum* is found to be 443 *μ*m^2^ and 186 *μ*m^2^, respectively. In good agreement the time-dependent model of *P. parvum* gives an area of 158 *μ*m^2^ (Fig. 5). From the model we calculate the disturbance distance as the equivalent spherical radius *r* of the disturbance zone for differently positioned power strokes with backwards pointing forces (Fig. 6b). The swimming speed is fixed to the maximum speed during the beat cycle of *P. parvum* and the disturbance distance *r*_∞_ for forces far from the cell body is used as reference. The disturbance distance for small force distances, i.e., for *R* < 3*a*, is found to be lowest for equatorial arrangements. For larger force distances, however, minimum detection risk occurs at angular force positions between the equator and the poles. The equatorial thrust force arrangement of *P. parvum* minimizes the disturbance zone with the intermediate force distance providing a trade-off between fast and quiet swimming.

### Prey capture on haptonema and cell

**Figure 1:**
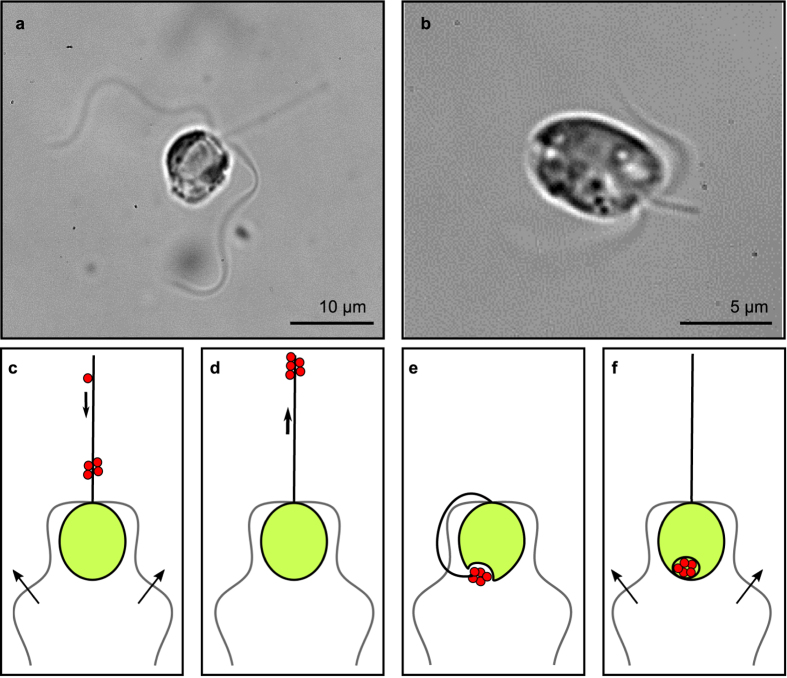
Individuals of the two studied haptophyte species and function of the haptonema. (**a**) *Prymnesium polylepis* and (**b**) *Prymnesium parvum*. (**c**–**f**) Sketch adapted from Kawachi and coworkers^8^. The haptophyte captures prey (red) on its haptonema while swimming and collects them at a specific aggregation point (**c**). While the flagella are paused, the aggregate is actively transported to the tip of the haptonema (**d**), which is bent towards the back of the cell (**e**), where the particles are engulfed (**f**). The purpose and means of the movement of prey towards the aggregation point are unknown.

**Figure 2:**
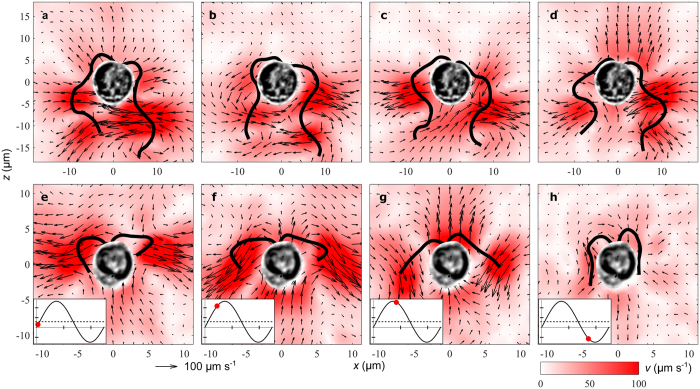
Instantaneous velocity fields measured around freely swimming individuals of the two haptophyte species during their beat cycles. (**a**–**d**) Velocity fields for *Prymnesium polylepis*, averaged over four beat cycles and (**e**–**h**) velocity fields for *Prymnesium parvum*, averaged over three beat cycles. Insets show measured cell velocity (ticks: 50 *μ*m s^−1^) as function of time (ticks: 10 ms). Instantaneous cell velocity (solid line, black) with beat cycle phase (filled circles, red) and average cell velocity (dashed line, black).

**Figure 3:**
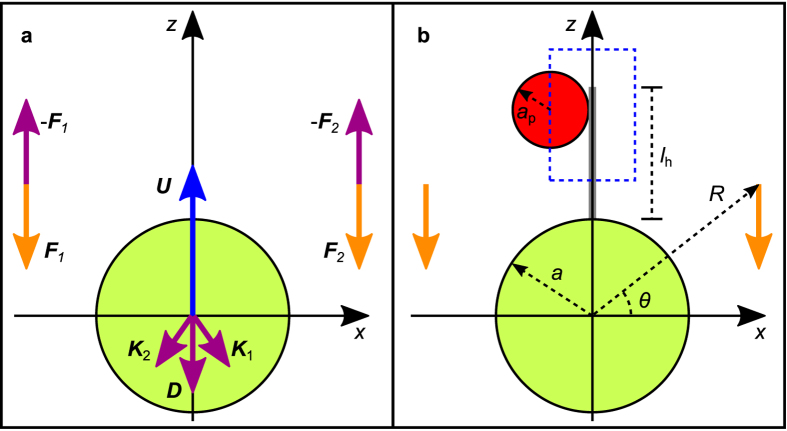
Biflagellate model and capture zone. (**a**) Model with a no-slip sphere (green) and two point forces (orange) representing the cell and the two flagella, respectively. The flagellate swims in the *z*-direction. The forces on the organism are shown as vectors in purple, i.e., the thrust forces −**F**_1_ and −**F**_2_, the forces **K**_1_ and **K**_2_ due to the flow produced by the point forces, and the Stokes drag **D** = −6*πμa***U** due to the translational motion of the cell with velocity **U** (vector, blue). (**b**) Haptophyte model with capture zone (dashed, blue) around the haptonema (grey) corresponding to a spherical prey (red).

**Figure 4:**
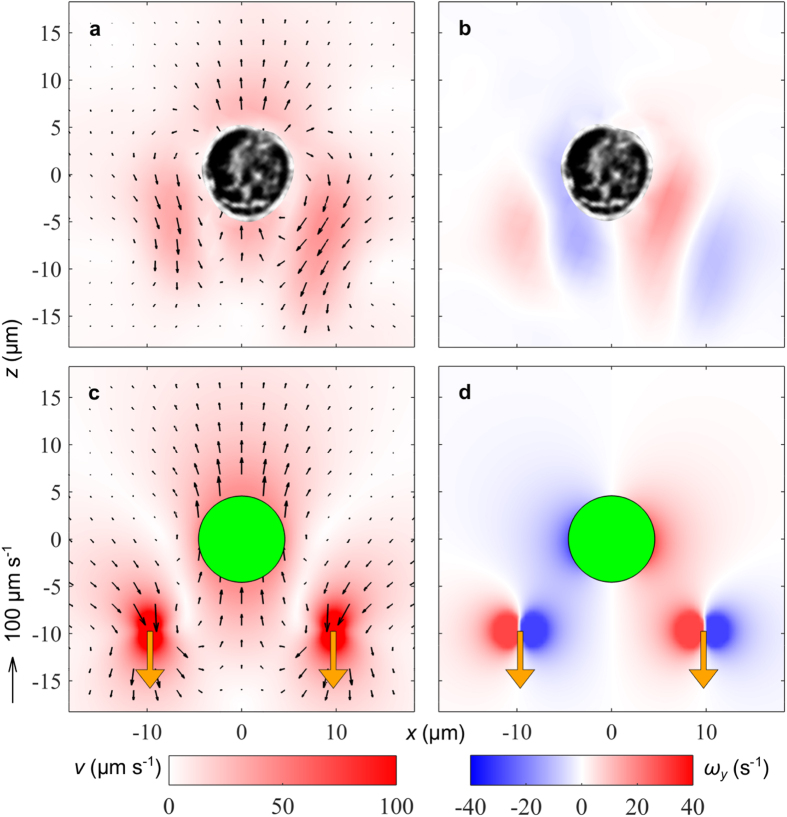
Measurements and biflagellate model results for the average velocity field and vorticity field for *Prymnesium polylepis*. (**a**,**b**) Measured velocity and vorticity, respectively, averaged over all frames in four beat cycles. (**c**,**d**) Modelled velocity and vorticity, respectively. The orange vectors show the location and the direction of the point forces on the water. The colour maps show the velocity magnitude *v* (**a**,**c**) and the vorticity component *ω*_*y*_ (**b**,**d**), i.e., counter-clockwise rotation in blue and clockwise rotation in red.

**Figure 5:**
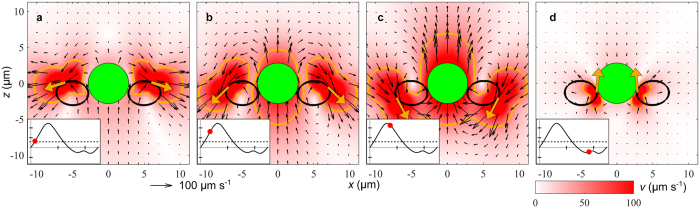
Time sequence of instantaneous velocity fields for the biflagellate model of *Prymnesium parvum*. The left-right symmetrically arranged point forces on the water (vectors, orange) move on elliptic trajectories (solid line, black) following the measured end points of the flagella. Insets show cell velocity (ticks: 50 *μ*m s^−1^) as function of time (ticks: 10 ms). Instantaneous cell velocity (solid line, black) with beat cycle phase (filled circles, red) and average cell velocity (dashed line, black). The area inside the contour (solid line, orange) is the cross-section in the *xz*-plane of the disturbance zone in which the flow speed exceeds the threshold speed 40 *μ*m s^−1^.

Haptophytes with a long haptonema such as *P. polylepis* use the haptonema for prey capture (Fig. 1c–f). How does the flagellar arrangement support this feeding and is it sufficient to fulfil the energy needs of the organism? The clearance rate is defined as the volume of ambient water cleared for prey per unit time^31^. The maximum clearance rate of eukaryotes that solely live from prey capture (heterotrophy) is found to be of the order of one million cell volumes per day^32^,^33^. Furthermore typical cell division rates of one per day suggest that at typical concentrations of available prey the empirically found maximum clearance rate is in fact needed in order to survive heterotrophically in the ocean^34^. This applies generally to marine heterotrophic microorganisms, and thus also to haptophytes if they are to survive on heterotrophy alone. Thus we here define the guideline daily amount (GDA) for heterotrophs as one million cell volumes per day. The presence of the haptonema is neglected in the model flow calculations, since an analytical estimate of the flow around the haptonema suggests, that it influences the flow only close to its surface (Methods). The advective clearance rate for capture treats the prey as passive tracers following the ambient flow. In the model it is estimated as the volume flow rate into a cylindrical capture zone with radius equal to the prey radius around the haptonema and into a spherical capture zone with radius equal to the cell radius plus the prey radius surrounding the cell (Fig. 3b). For the calculation, the prey particles are treated as spherical particles with radius *a*_p_ = 1 *μ*m attaching with 100% efficiency to the haptonema or cell upon touch. Our choice of prey size is based on the observations on prey selectivity of the related species *Haptolina hirta*, for which prey in the range 0.3–4.1 *μ*m in diameter were ingested with a favoured prey of length 2.5 *μ*m 8. The thrust force is held constant at the value for the average flow of *P. polylepis* (Fig. 4).

**Figure 6:**
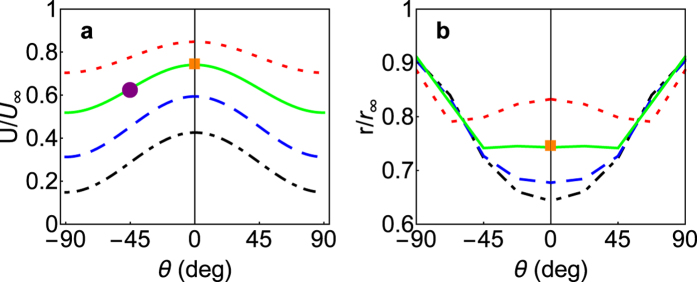
Normalized swimming speed and flow disturbance distance for different flagellar arrangements. Angular force position *θ* and radial force position: *R* = 1.5*a* (dotted-dashed lines, black), *R* = 2*a* (dashed lines, blue), *R* = 3*a* (solid lines, green), and *R* = 5*a* (dotted lines, red). (**a**) Modelled swimming speed (lines) and estimated time-averaged values for *Prymnesium polylepis* (filled circle, purple) and *Prymnesium parvum* (filled square, orange). A maximum of the swimming speed is found for equatorially placed, far-away forces (*θ* = 0 deg, *R* large). (**b**) The disturbance distance modelled for the maximum thrust force magnitude of *P. parvum* (lines) and the estimated value for the power stroke of *P. parvum* (filled square, orange). Maximum disturbance is found for force arrangements at the poles (*θ* = ±90 deg) and for *R* < 3*a* the smallest disturbance distance is found for equatorial arrangements (*θ* = 0 deg).

**Figure 7:**
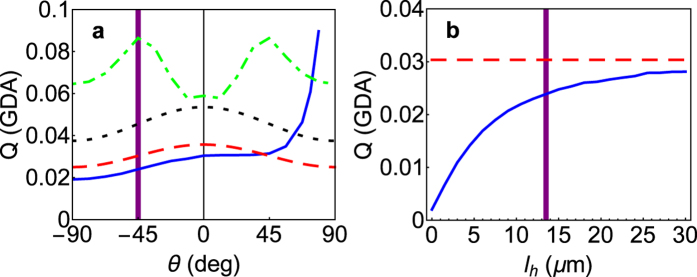
Advective clearance rate on haptonema and cell of *Prymnesium polylepis*. (**a**) The clearance rate on the haptonema (solid line, blue) and cell (dotted-dashed line, green) as function of the angular force position with approximations 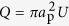 from ballistic encounter on the haptonema (dashed line, red) and 
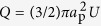
 from capture on a towed sphere (dotted line, black), respectively. The clearance rate on the haptonema is largest for puller arrangements (*θ* = 90 deg) and on the cell it is maximal for flagellar forces arranged at *θ* = ±45 deg. (**b**) The clearance rate on the haptonema (solid line, blue) as function of haptonema length with ballistic approximation (dashed line, red). Observed force position (a) and haptonema length of (b)*P. polylepis* are indicated (vertical lines, purple). The guideline daily amount (GDA) is defined as one million cell volumes per day.

For pusher arrangements the clearance rate on the haptonema follows the estimate of ballistic encounter of passive prey on the moving haptonema, which is calculated as the projected area of the encounter zone on the swimming direction times the swimming speed, i.e., 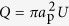. Thus the advective clearance rate on the haptonema has a local maximum at an equatorial force position due to the maximum in swimming speed (Figs 6a and 7a). For puller arrangements the velocity of the feeding current towards the haptonema exceeds the swimming speed and leads to a high increase of the clearance rate, suggesting an optimum arrangement with forces acting right next to the haptonema. On the other hand, the calculated clearance rate on the cell body does not distinguish between puller and pusher arrangements and has maxima for forces positioned at *θ* = ±45 deg. The approximation 
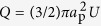
 for small 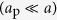 and passive prey is estimated from the creeping flow past a towed sphere as the volume flow rate within a stream-tube that encloses the sphere with the closest distance *a*_p_^31^,^35^,^36^. This formula matches only for forces close to the cell equator and does not capture the two maxima. *Prymnesium polylepis* with mean forces positioned approximately 45 deg behind the cell body does not seem to be optimized for advective prey capture on the haptonema, but apparently for capture on the cell body. The cell surface, which is lined with spine scales, has, however, not been confirmed to be able to capture and transport small prey to the ingestion site on the back. The advective clearance rate of *P. polylepis* increases and eventually saturates with increasing haptonema length, and the observed mean haptonema length ensures a clearance rate near the asymptotic value (Fig. 7b and Table 2).

To what extent does the motility of the prey contribute to capture on the haptonema? For the following estimates we focus on encounter purely by random motion without advection. Motile prey such as swimming bacteria are encountered ballistically, if their run length, i.e., the length of their straight trajectory segments, is larger than the size of the capture zone^37^. The ballistic clearance rate for a stationary capture zone of surface area *S* collecting small randomly moving prey with swimming speed *u* and uniformly distributed directions can be calculated as *Q* = (1/4)*Su* (Methods). This simple formula provides a generalization of the well-known formula, *Q* = *πa*^2^*u*, for spherical capture zones^38^,^39^,^40^. To calculate the ballistic clearance rate the haptonema surface is calculated as that of a cylinder, i.e., *S* = 2*πa*_p_(*l*_h_ + *a*_p_) with the haptonema length *l*_h_ and the prey radius *a*_p_. If the run length of the motile prey is smaller than the size of the capture zone, particle capture creates a significant concentration gradient and encounter is best considered as effective diffusion with the diffusion coefficient *D*_eff_ = *u*^2^*τ*/3 depending on *u* the prey speed and *τ* the run time^41^. This process is considerably faster than Brownian diffusion of passive particles with *D* = *k*_B_*T*/(6*πμa*_p_), where *k*_B_*T* is the Boltzmann energy at 16 °C. For both diffusive processes the haptonema is treated as a slender spheroid resulting in the clearance rate *Q* = 2*πDl*_h_/ln(*l*_h_/*a*_p_) (Table 2)^42^. Our estimates of advective, diffusive, and ballistic clearance rates of *P. polylepis* show that all contributions are considerably lower than the estimated guideline daily amount for heterotrophs. For micrometer-sized prey Brownian diffusion is negligible. Encounters of motile prey, both ballistic and effective diffusive, give the highest estimated clearance rates, however, still ten times lower than the GDA (Table 2).

## Discussion

We have shown that our analytical model can be used to represent near cell flows around freely swimming biflagellates with different flagellar arrangements. With the model we identified trade-offs between equatorial flagellar arrangements that favour fast and quiet swimming as opposed to puller arrangements that favour feeding. The optimal force distance is far away to swim fast and close to the cell body in order to create the least flow disturbance. The clearance rate estimates for the example of *P. polylepis* result in values which are too low to ensure survival in the pelagic realm, and we therefore argue that photosynthesis and nutrient uptake are likely to be essential for the survival of this species and potentially other mixotrophic biflagellates.

The time-averaged thrust force magnitudes per unit flagellum length in the two biflagellates were found to be approximately equal (Table 1). *Prymnesium parvum* was shown to have a favourable equatorial beat pattern and intermediate force distances that appear to make a compromise between fast and quiet swimming. *Prymnesium polylepis*, in contrast, was, based on its time-averaged force arrangement, not found to be particularly optimal, neither for swimming nor for advective feeding even though this species is dependent on encountering small prey. The low advective contribution to prey capture is one possible reason why the flagellar arrangement in *P. polylepis* is not defined by the optimum for steady advective feeding. Furthermore, the flagella and the highly time-varying flows around them could hinder prey capture, if the flagella were in a puller arrangement positioned close to the haptonema.

Another aspect of prey capture in *P. polylepis* is the design of the haptonema that is optimized as a long slender structure favourable for prey encounter, but with physical limitations given by the cost of production, stability, flow resistance, and the ability to reach the ingestion site at the back end of the cell. The created feeding flow does not only support the motion of prey towards but also along the haptonema towards the aggregation point close to the cell body (Fig. 1c). The Stokes drag on particles of 1 *μ*m radius moving with the local flow velocity decreases to around 0.4 pN at the distance of 5 *μ*m from the cell body, reported as the typical location of the aggregation point^8^. The transport of captured prey along the haptonema towards the aggregation point can therefore be purely due to the flow created by the flagellar beat if the friction on the haptonema is not larger than this drag. We speculate that the positioning of the prey at the aggregation point allows the haptophyte to hold on to the prey with the least effort without blocking the capture of additional prey.

Some of the above predictions can readily be applied to other biflagellates. For example the 2–3 times speed difference between the two swimming modes of *C. reinhardtii* could, according to our model analysis, be due to the difference in angular force arrangements between the ciliary puller beat and the undulatory pusher beat^22^. Furthermore the time-varying biflagellate model with moving point forces implies that an approximately four times larger force is needed to propel the unsteady swimmer *P. parvum*, than the time-averaged model suggests. A similar numerical factor has earlier been found by comparison of power dissipation in time-resolved and time-averaged flows around *C. reinhardtii*^26^. As an outlook the model can be generalized to fit other microswimmers with different numbers and arrangements of appendages, further taking into account body rotation using the torque balance. It will in particular be relevant to examine how purely heterotrophic flagellates acquire sufficient nutrition^12^.

## Methods

### Cultures and observations

Cultures were grown in B1-medium (non-axenic) with a salinity of 32 at 20 °C. They were subjected to 100 *μ*mol photons m^−2^ s^−1^ on a 12:12 h light:dark cycle. Observations of swimming haptophyte cells were made using an Olympus IX71 inverted microscope equipped with a 100 × DIC objective, and in some cases an additional 2 × magnifying lens. Recordings were made using a Phantom V210 high-speed (1000 fps for *P. polylepis*, 500 fps for *P. parvum*), high-resolution (1280 × 800 pixels) digital video camera. Fields of view were 0.26 mm × 0.16 mm for *P. polylepis* and 0.13 mm × 0.08 mm for *P. parvum*. The organisms swam in 10 mm × 10 mm × 0.5 mm chambers mounted on a microscope slide with silicone grease. The microscope was focused at the full working distance of the lens (150 *μ*m) from the cover glass to limit wall effects. Observations were made in an air-conditioned room set to 16 °C.

### Flow measurements

Flow fields were measured with micro particle image velocimetry. The medium was seeded with neutrally buoyant, polymer spheres with diameter *d* = 300 nm. The focal depth of the objective of approximately 1 *μ*m defined the thickness of the observation plane. Organisms were masked using ImageJ 1.46r prior to analysis. We used a multi-pass algorithm in DaVis PIV software 8.0.6 (Lavision GmbH, Göttingen, Germany) with decreasing size of the interrogation windows, with a final window size of 32 × 32 pixels with 75% overlap. There were on average *N*_p_ = 16 and *N*_p_ = 4 particles in each interrogation window for *P. polylepis* and *P. parvum*, respectively. The flow speed resolution limit due to Brownian motion of seeding particles was estimated as 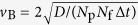 with *D* = *k*_B_*T*/(3*πμd*), the time resolution Δ*t*, and *N*_f_ the number of averaged frames^43^. For the time-resolved flow fields we calculated *v*_B_ = 8.8 *μ*m s^−1^ for *P. polylepis* with *N*_f_ = 4 and *v*_B_ = 14.4 *μ*m s^−1^ for *P. parvum* with *N*_f_ = 3.

### Flow profile around the haptonema

To estimate the effect of the haptonema on the flow in front of the cell body of *P. polylepis* we calculated the Stokes flow past a prolate no-slip spheroid with the constant far field flow velocity *U* in the length direction [44, p. 154f.]. With the half-major axis 6.75 *μ*m and half-minor axis 0.1 *μ*m we found that the flow velocity is decreased by one half on a spheroidal stream surface around the haptonema with a distance 0.17 *μ*m from the tip and 1.41 *μ*m from the middle of the haptonema.

### Calculation of ballistic clearance rates

We derived a simple formula that allows immediate calculation of the clearance rate as *Q* = (1/4)*Su* for any stationary capture zone with surface area *S* when the prey are uniformly distributed point particles ballistically moving with speed *u* with equal probability in all directions [39, p. 179, eq. (5–32)]. We obtained this result by calculating the clearance rate per unit surface area as 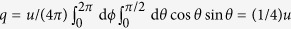 and multiplying by the total surface area, i.e., *Q* = *Sq*.

## Additional Information

**How to cite this article**: Dölger, J. *et al*. Swimming and feeding of mixotrophic biflagellates. *Sci. Rep.* **7**, 39892; doi: 10.1038/srep39892 (2017).

**Publisher's note:** Springer Nature remains neutral with regard to jurisdictional claims in published maps and institutional affiliations.

## Supplementary Material

Supplementary Video S1

Supplementary Video S2

Supplementary Video S3

Supplementary Video S4

Supplementary Information

## Figures and Tables

**Table 1:** Morphology, flagellar dynamics, and swimming speed of *Prymnesium polylepis* and *Prymnesium parvum*.

	P. polylepis	P. parvum
Cell length (*μ*m)	9.1 ± 0.8	7.2 ± 0.3
Cell width (*μ*m)	6.8 ± 0.4	5.4 ± 0.5
ESD of cell 2*a(μ*m)	9	6
Haptonema length *l*_h_ (*μ*m)	13.5 ± 1.3	3.4 ± 0.6
Length of flagella (*μ*m)	28	10
Beat period of flagella (ms)	30	25
Average swimming speed *U(μ*m s^−1^)	45	30
Average point force magnitude *F* (pN)	3	1
Radial force position *R(μ*m)	14(=3*a*)	8(=3*a*)
Angular force position *θ* (deg)	−45	0

The values for cell length, cell width, and haptonema length are averages based on five individuals (mean ± SD), and the values for equivalent spherical diameter of the cell (ESD), length and beat period of the flagella, swimming speed, point force magnitude and position are based on two closely studied individuals. The time-averaged force positions were roughly estimated by visual comparison of the time-averaged flow fields from experiment and model, and the point force magnitudes were calculated using equation (3) with the estimated time-averaged force positions and the measured time-averaged swimming velocities.

**Table 2:** Advective, ballistic, and diffusive clearance rate estimates for non-motile and motile prey of *Prymnesium polylepis*.

Encounter model	Example prey	ESD (*μ*m)	*u(μ*m s^−1^)	*τ* (s)	*λ(μ*m)	*Q(GDA*)
Advective (non-motile prey)	Spherical tracers	2	0	/	0	0.024
Brownian diffusive (non-motile prey)	Spherical tracers	2	0	/	0	0.002
Ballistic (motile prey)	*Microscilla furvescens* (M58792)	1.7	32	1.4	46	0.132
Effective diffusive (motile prey)	Marine bacterium TW-3 (AY028198)	1.2	44	0.04	2	0.151

Equivalent spherical diameter (ESD), swimming speed *u*, run time *τ*, and run length *λ* of the prey^37^. The clearance rate *Q* on the haptonema in units of the guideline daily amount (GDA), defined as one million cell volumes per day.
