# Supplementary material for: Swimming and feeding of mixotrophic biflagellates
Source: Sci Rep. 2017 Jan 5;7:39892. doi: 10.1038/srep39892 (PMC5215566; doi:10.1038/srep39892)
Supplement: Supplementary Information [file srep39892-s5.pdf]

Swimming and feeding of mixotrophic biflagellates
# - Supplementary Information

Julia Dölger, Lasse Tor Nielsen, Thomas Kiørboe, and Anders Andersen

Supplementary Videos

Supplementary Video S1: *Prymnesium polylepis*, 100 times slowed down. The cell in this video is stuck to the microscope slide and thus not freely swimming. Figure 1 a shows a still image of this movie.

Supplementary Video S2: Freely swimming *Prymnesium parvum*, 50 times slowed down. Figure 1 b shows a still image of this movie.

Supplementary Video S3: Freely swimming *Prymnesium polylepis* surrounded by polystyrene particles used for μPIV, 100 times slowed down (cf. Fig. 2 a-d and Fig. 4).

Supplementary Video S4: Freely swimming *Prymnesium parvum* surrounded by polystyrene particles used for μPIV, 50 times slowed down (cf. Fig. 2 e-h and Supplementary Fig. S5 online)**.**

Supplementary Figure

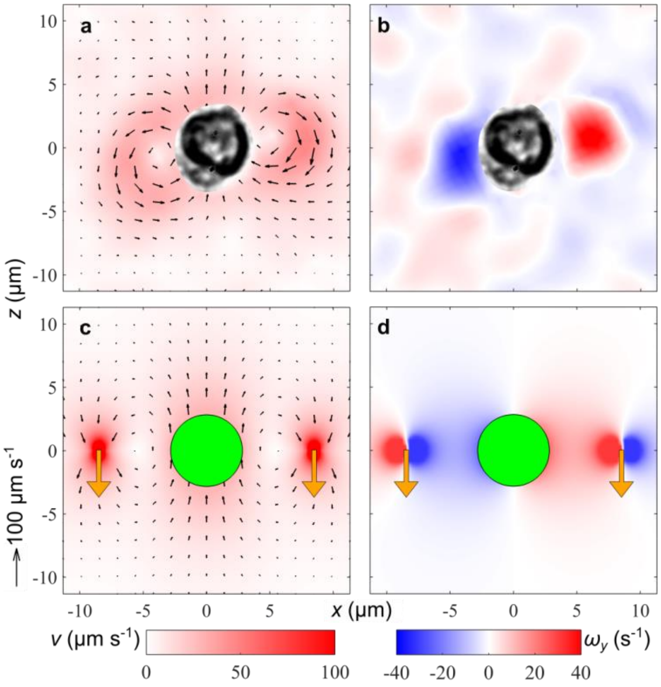

Supplementary Figure S5: Measurements and biflagellate model results for the average velocity field and vorticity field for *Prymnesium parvum*. (a-b) Measured velocity and vorticity, respectively, averaged over all frames in three beat cycles. (c-d) Modelled velocity and vorticity, respectively. The orange vectors show the location and the direction of the point forces on the water. The colour maps show the velocity magnitude v (a,c) and the vorticity component ω_y (b,d), i.e., counter-clockwise rotation in blue and clockwise rotation in red.
